# Intranasal fusion inhibitory lipopeptide prevents direct-contact SARS-CoV-2 transmission in ferrets

**DOI:** 10.1126/science.abf4896

**Published:** 2021-02-17

**Authors:** Rory D. de Vries, Katharina S. Schmitz, Francesca T. Bovier, Camilla Predella, Jonathan Khao, Danny Noack, Bart L. Haagmans, Sander Herfst, Kyle N. Stearns, Jennifer Drew-Bear, Sudipta Biswas, Barry Rockx, Gal McGill, N. Valerio Dorrello, Samuel H. Gellman, Christopher A. Alabi, Rik L. de Swart, Anne Moscona, Matteo Porotto

**Affiliations:** 1 Department of Viroscience, Erasmus MC, Rotterdam, Netherlands.; 2 Department of Pediatrics, Columbia University Vagelos College of Physicians and Surgeons, New York, NY, USA.; 3 Center for HostPathogen Interaction, Columbia University Vagelos College of Physicians and Surgeons, New York, NY, USA.; 4 Department of Experimental Medicine, University of Campania Luigi Vanvitelli, Caserta, Italy.; 5 Department of Biomedical Engineering, Politecnico di Milano, Milan, Italy.; 6 Digizyme Inc., Brookline, MA, USA.; 7 Department of Physiology and Cellular Biophysics, Columbia University Vagelos College of Physicians and Surgeons, New York, NY, USA.; 8 Robert Frederick Smith School of Chemical and Biomolecular Engineering, Cornell University, Ithaca, NY, USA.; 9 Center for Molecular and Cellular Dynamics, Department of Biological Chemistry and Molecular Pharmacology, Harvard Medical School, Boston, MA, USA.; 10 Department of Chemistry, University of WisconsinMadison, Madison, WI, USA.; 11 Department of Microbiology and Immunology, Columbia University Vagelos College of Physicians and Surgeons, New York, NY, USA.

## Abstract

Containment of the COVID-19 pandemic requires reducing viral transmission. Severe acute respiratory syndrome coronavirus 2 (SARS-CoV-2) infection is initiated by membrane fusion between the viral and host cell membranes, which is mediated by the viral spike protein. We have designed lipopeptide fusion inhibitors that block this critical first step of infection and, on the basis of in vitro efficacy and in vivo biodistribution, selected a dimeric form for evaluation in an animal model. Daily intranasal administration to ferrets completely prevented SARS-CoV-2 direct-contact transmission during 24-hour cohousing with infected animals, under stringent conditions that resulted in infection of 100% of untreated animals. These lipopeptides are highly stable and thus may readily translate into safe and effective intranasal prophylaxis to reduce transmission of SARS-CoV-2.

## Halting transmission

The severe acute respiratory syndrome coronavirus 2 (SARS-CoV-2) spike (S) glycoprotein binds to host cells and initiates membrane fusion and cell infection. This stage in the virus life history is currently a target for drug inhibition. De Vries *et al.* designed highly stable lipoprotein fusion inhibitors complementary to a conserved repeat in the C terminus of S that integrate into host cell membranes and inhibit conformational changes in S necessary for membrane fusion. The authors tested the performance of the lipoproteins as a preexposure prophylactic in a ferret-to-ferret transmission study. Intranasal administration of the peptide 2 days before cohousing with an infected ferret for 24 hours completely protected animals in contact from infection and showed efficacy against mutant viruses. Because ferrets do not get sick from SARS-CoV-2, disease prevention could not be tested in this model.

*Science*, this issue p. 1379

## Teaser

A dimeric form of a SARS-CoV-2derived lipopeptide is a potent inhibitor of fusion and infection in vitro and transmission in vivo.

**Fig. 1:**
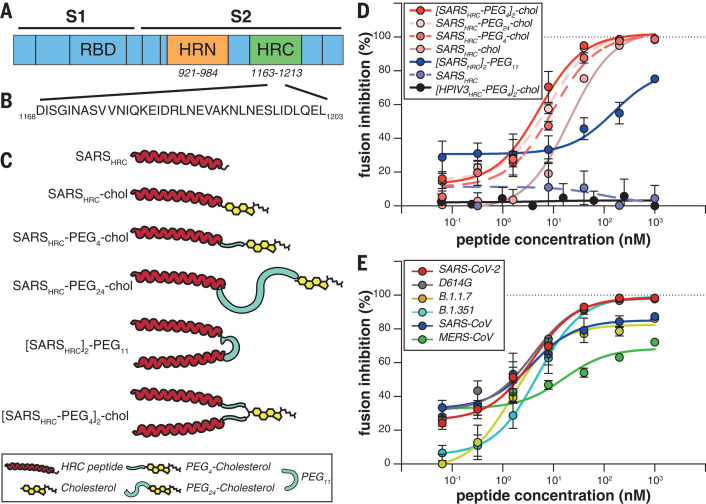
Peptide-lipid conjugates that inhibit SARS-CoV-2 spike (S)mediated fusion. (**A**) The functional domains of SARS-CoV-2 S protein, the receptor binding domain (RBD) and heptad repeats (HRN and HRC), are indicated. (**B**) Sequence of the peptides derived from the HRC domain of SARS-CoV-2 S. Single-letter abbreviations for the amino acid residues are as follows: A, Ala; C, Cys; D, Asp; E, Glu; F, Phe; G, Gly; H, His; I, Ile; K, Lys; L, Leu; M, Met; N, Asn; P, Pro; Q, Gln; R, Arg; S, Ser; T, Thr; V, Val; W, Trp; and Y, Tyr. (**C**) Monomeric and dimeric forms of lipid-tagged SARS-CoV-2 inhibitory peptides that were assessed in cellcell fusion assays. (**D**) Cellcell fusion assays with different inhibitory peptides. The percentage inhibition is shown for six different SARS-CoV-2specific peptides and a control HPIV-3specific peptide at increasing concentrations. Percent inhibition was calculated as the ratio of the relative luminescence units in the presence of a specific concentration of inhibitor (X) and the relative luminescence units in the absence of inhibitor, corrected for background luminescence. Percent inhibition = 100 [1 (luminescence at X background)/(luminescence in absence of inhibitor background)]. The difference between the results for [SARS_HRC_-PEG_4_]_2_-chol and SARS_HRC_-PEG_4_-chol lipopeptides was statistically significant [two-way analysis of variance (ANOVA), *P* < 0.0001]. (**E**) Fusion inhibitory activity of [SARS_HRC_-PEG_4_]_2_-chol peptide against emerging SARS-CoV-2 S variants, MERS-CoV-2 S, and SARS-CoV S. Data in (D) and (E) are means standard error of the mean (SEM) from three separate experiments, with the curve representing a four-parameter dose-response model.

Infection by severe acute respiratory syndrome coronavirus 2 (SARS-CoV-2) requires membrane fusion between the viral envelope and the host cell, at either the cell surface or the endosomal membrane. The fusion process is mediated by the viral transmembrane spike glycoprotein (S). Upon viral attachment or uptake, host factors trigger large-scale conformational rearrangements in S, including a refolding step that leads directly to membrane fusion and viral entry (*1*,*3*). Peptides corresponding to the highly conserved heptad repeat (HR) (Fig. 1A) domain at the C terminus of the S protein (HRC peptides) (Fig. 1B) can prevent this refolding and inhibit fusion, thereby preventing infection (*4*,*8*). The HRC peptides form six-helix bundle-like assemblies with the extended intermediate form of the S protein trimer, disrupting the structural rearrangement of S that drives membrane fusion (*4*) (see also movie S1).

Our approach in designing SARS-CoV-2 S-specific fusion inhibitors builds on our previous work that demonstrated that lipid conjugation of HRC-derived inhibitory peptides markedly increases antiviral potency and in vivo half-life (*9*, *10*). These peptides successfully inhibit human parainfluenza virus type 3 (HPIV-3), measles virus, influenza virus, and Nipah virus infection (*9*, *11*,*13*). Furthermore, the combination of dimerization and lipopeptide integration into cell membranes protects the respiratory tract and prevents systemic lipopeptide dissemination (*14*). Lipid-conjugated peptides administered intranasally to animals reached high concentrations both in the upper and lower respiratory tract, and the lipid could be designed to modulate the extent of transit from the lung to the blood and organs (*9*, *14*). We propose a SARS-CoV-2specific lipopeptide as a candidate antiviral for preexposure and early postexposure prophylaxis for SARS-CoV-2 transmission in humans.

We recently described a monomeric SARS-CoV-2 HRC-lipopeptide fusion inhibitor (*4*) against SARS-CoV-2 with in vitro and ex vivo efficacy superior to previously described HRC-derived fusion inhibitory peptides (*6*, *7*). To further improve antiviral potency, we compared monomeric and dimeric HRC-peptide derivatives (Fig. 1C). Figure 1D shows antiviral potency in a quantitative cellcell fusion assay of four monomeric and two dimeric SARS-CoV-2 S-derived 36amino acid HRC peptides (Fig. 1B; see also figs. S1A and S3 for structure and characterization), with or without appended cholesterol. Dimerization increased the peptide potency for both nonlipidated peptides and their lipidated counterparts (Fig. 1D). A dimeric cholesterol-conjugated lipopeptide based on the HPIV-3 F protein HRC domain, used as a negative control, did not inhibit fusion at any concentration tested (black line in Fig. 1D; see fig. S1, B and C, for additional negative controls). Among the monomeric lipopeptides, the peptide bearing 24 molecules of polyethylene glycol (PEG_24_) was most potent. The dimeric cholesterol-conjugated peptide ([SARS_HRC_-PEG_4_]_2_-chol; red line in Fig. 1D) was the most potent lipopeptide against SARS-CoV-2 among our panel. This peptide also robustly inhibited fusion mediated by the S proteins of several emerging SARS-CoV-2 variants [including D614G, the variant bearing the Asp^614^Gly mutation (*15*)], the recent variants of concern B.1.1.7 and B.1.351 (*16*, *17*), and the S protein of SARS-CoV and Middle East respiratory syndrome coronavirus (MERS-CoV) (Fig. 1E). Proposed anchoring of the dimeric lipopeptide in the host cell membrane and interactions with the viral S protein are shown in fig. S2 and movie S1. Collectively, these data suggest that the [SARS_HRC_-PEG_4_]_2_-chol lipopeptide is equipped to combat an evolving pandemic.

**Fig. 2:**
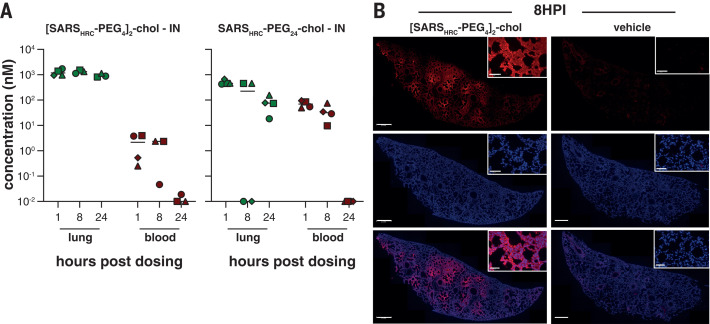
Biodistribution of [SARS_HRC_-PEG_4_]_2_-chol and SARS_HRC_-PEG_24_ peptides after intranasal administration to mice. (**A**) The concentration of lipopeptides (*y* axis) was measured by ELISA in lung homogenates and plasma samples (*n* = 4 mice, with the exception of [SARS_HRC_-PEG_4_]_2_-chol IN, for which *n* = 3 at 8 and 24 hours, and *n* = 1 for vehicle treatment). Median is indicated by horizontal bar. (**B**) Lung sections of [SARS_HRC_-PEG_4_]_2_-chol-treated (or vehicle-treated) mice were stained with anti-SARS-HRC antibody (red) confirming broad distribution of [SARS_HRC_-PEG_4_]_2_-chol in the lung (8 hours post inoculation, 8HPI). Scale bar, 500 m in lung tile scan and 50 m in magnification; representative images and a full tile scan are shown. Nuclei were counterstained with 4,6-diamidino-2-phenylindole (blue).

For other enveloped respiratory viruses, we previously showed that both ex vivo and in vivo dimeric lipopeptides (administered intranasally) displayed increased retention in the respiratory tract compared with monomeric compounds (*14*). In this study, we compared local and systemic biodistribution of our most potent monomeric and dimeric lipopeptides (SARS_HRC_-PEG_24_-chol and [SARS_HRC_-PEG_4_]_2_-chol) at 1, 8, and 24 hours after intranasal inoculation or subcutaneous injection in humanized K18 hACE2 mice (Fig. 2 and fig. S4). The two lipopeptides reached a similar lung concentration at 1 hour after intranasal administration (~1 to 2 M). At 8 and 24 hours, the dimeric [SARS_HRC_-PEG_4_]_2_-chol lipopeptide remained at high concentrations in the lung with minimal entry into the blood, but the monomeric peptide entered the circulation and the lung concentration decreased (Fig. 2A). The dimeric [SARS_HRC_-PEG_4_]_2_-chol lipopeptide was distributed throughout the lung after intranasal administration (Fig. 2B). A cellular toxicity (MTT) assay in primary human airway epithelial cells showed minimal toxicity even after 6 days at the highest concentrations tested (<20% at 100 M) and no toxicity at its 90% maximal inhibitory concentration (IC_90_) (~35 nM) (fig. S5). The longer respiratory tract persistence of [SARS_HRC_-PEG_4_]_2_-chol, in concert with its in vitro efficacy, led us to advance this dimeric lipopeptide.

**Fig. 3:**
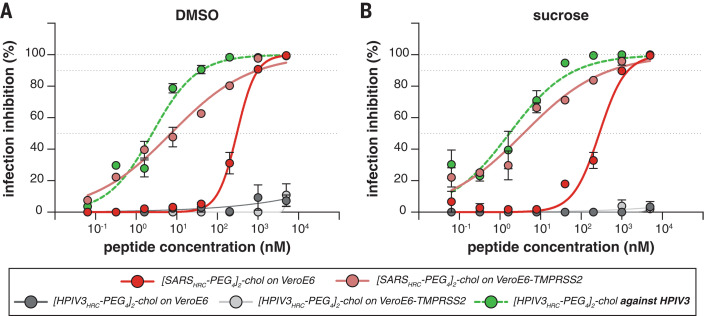
Inhibition of infectious SARS-CoV-2 entry by [SARS_HRC_-PEG_4_]_2_-chol and [HPIV-3_HRC_-PEG_4_]_2_-chol peptides. (**A** and **B**) The percentage inhibition of infection is shown on VeroE6 and VeroE6-TMPRSS2 cells with increasing concentrations of [SARS_HRC_-PEG_4_]_2_-chol (red lines) and [HPIV-3_HRC_-PEG_4_]_2_-chol (gray lines). DMSO-formulated (A) and sucrose-formulated (B) stocks were tested side by side. Mean SEM of triplicates is shown; dotted lines show 50% and 90% inhibition. Additionally, the potency of [HPIV-3_HRC_-PEG_4_]_2_-chol was confirmed by inhibition of infectious HPIV-3 entry (dotted green lines, performed on Vero cells).

The lead peptide, [SARS_HRC_-PEG_4_]_2_-chol, was assessed for its ability to block entry of SARS-CoV-2 in VeroE6 cells or VeroE6 cells overexpressing transmembrane serine protease 2 (TMPRSS2), one of the host factors thought to facilitate viral entry at the cell membrane (*2*). Whereas viral fusion in VeroE6 cells predominantly occurs after endocytosis, the virus enters TMPRSS2-overexpressing cells by fusion at the cell surface, reflecting the entry route in airway cells (*18*). This difference is highlighted by chloroquines effectiveness against SARS-CoV-2 infection in Vero cells but its failure in TMPRSS2-expressing Vero cells and the human lung (*19*). The [SARS_HRC_-PEG_4_]_2_-chol peptide dissolved in an aqueous buffer containing 2% dimethyl sulfoxide (DMSO) inhibited virus entry after 8 hours with a half-maximal inhibitory concentration (IC_50_) of ~300 nM in VeroE6 and ~5 nM in VeroE6-TMPRSS2 cells (Fig. 3A). To strengthen translational potential toward human use, the lipopeptide was reformulated in sucrose instead of DMSO, resulting in equivalent in vitro potency (Fig. 3B). A control dimeric fusion-inhibitory lipopeptide directed against HPIV-3 blocked infection by HPIV-3 but did not inhibit SARS-CoV-2 infection. The in vitro efficacy data are summarized in table S1.

Ferrets are an ideal model for assessing respiratory virus transmission, either by direct contact or by aerosol transmission (*20*, *21*). Mustelids are highly susceptible to infection with SARS-CoV-2, as also illustrated by frequent COVID-19 outbreaks at mink farms. Direct-contact transmission of SARS-CoV in ferrets was demonstrated in 2003 (*22*), and both direct-contact and airborne transmission of SARS-CoV-2 have been shown in ferrets (*20*, *23*). Direct-contact transmission in the ferret model is highly reproducible (100% transmission from donor to acceptor animals), but ferrets display limited clinical signs. After infection via direct inoculation or transmission, SARS-CoV-2 can readily be detected in and isolated from the throat and nose, and viral replication leads to seroconversion.

To assess the efficacy of [SARS_HRC_-PEG_4_]_2_-chol in preventing SARS-CoV-2 transmission, nave ferrets were dosed prophylactically with the lipopeptide before being cohoused with SARS-CoV-2infected ferrets. In this setup, transmission via multiple routes can theoretically occur (aerosol, orofecal, and scratching or biting), and ferrets are continuously exposed to infectious virus during the period of cohousing, providing a stringent test for antiviral efficacy. The study design is shown in fig. S6. Three donor ferrets (gray in fig. S6) were inoculated intranasally with 5 10^5^ 50% tissue culture infective dose (TCID_50_) SARS-CoV-2 on day 0. Twelve recipient ferrets housed separately were treated by nose drops with a mock preparation or [SARS_HRC_-PEG_4_]_2_-chol peptide (red and green, respectively, in fig. S6) 1 and 2 days post inoculation (dpi) of the donor animals. The [SARS_HRC_-PEG_4_]_2_-chol peptides for intranasal administration were dissolved to a concentration of 6 mg/ml in an aqueous buffer containing 2% DMSO, and ferrets were administered a final dose of 2.7 mg/kg of body weight (450 l, divided equally in both nostrils). Peptide stocks and working dilutions had similar IC_50_ values, confirming that peptide-treated ferrets were dosed daily with comparable amounts (fig. S7, A and B). Six hours after the second treatment on 2 dpi, one infected donor ferret [highly positive for SARS-CoV-2, as indicated by reverse transcriptionquantitative polymerase chain reaction (RT-qPCR)] was cohoused with four nave recipient ferrets (two mock-treated and two peptide-treated). After a 24-hour transmission period in three separate, negatively pressurized HEPA-filtered Animal Biosafety Level 3 (ABSL3)isolator cages, cohousing was stopped and donor, mock-treated, and peptide-treated ferrets were rehoused as separate groups. Additional [SARS_HRC_-PEG_4_]_2_-chol peptide treatments were given to recipient animals on 3 and 4 dpi.

**Fig. 4:**
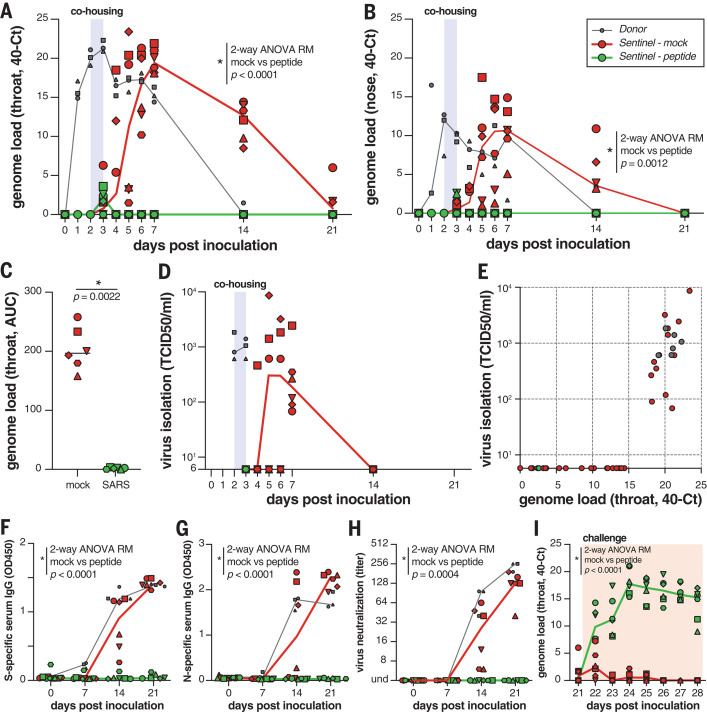
[SARS_HRC_-PEG_4_]_2_-chol prevents SARS-CoV-2 transmission in vivo. (**A** and **B**) Viral loads detected in throat (A) and nose (B) swabs by RT-qPCR. (**C**) Comparison of the AUC from genome loads reported in (B) for mock- and peptide-treated sentinels. (**D**) Viral loads detected in throat swabs by virus isolation on VeroE6. (**E**) Correlation between viral loads in the throat as detected via RT-qPCR and virus isolation. Presence of anti-S (**F**) or anti-N (**G**) antibodies was determined by IgG ELISA assay. Presence of neutralizing antibodies was determined in a virus neutralization assay (**H**). Virus neutralizing antibodies are displayed as the end-point serum dilution factor that blocks SARS-CoV-2 replication. Direct inoculation of peptide-treated or mock-treated animals with SARS-CoV-2 led to productive infection in only the previously peptide-treated animals (**I**), in the absence of S-specific, N-specific, and neutralizing antibodies. Donor animals shown in gray, mock-treated animals in red, peptide-treated animals in green. Symbols correspond to individual animals (defined in fig. S6). Line graphs in (A), (B), (D), and (F) to (I) connect the median of individual animals per time point. Mock- and peptide-treated groups were compared using two-way ANOVA repeated measures [(A), (B), and (F) to (I)] or Mann-Whitney test (C).

The viral loads (detection of viral genomes using RT-qPCR) for directly inoculated donor animals (gray), mock-treated recipient animals (red), and lipopeptide-treated recipient animals (green) are shown in Fig. 4, A and B. All directly inoculated donor ferrets were productively infected, as shown by SARS-CoV-2 genome detection in throat and nose swabs, and efficiently and reproducibly transmitted the virus to all mock-treated acceptor ferrets (Fig. 4, A and B, red curves). Productive SARS-CoV-2 infection was not detected in the throat or nose of any of the peptide-treated recipient animals (Fig. 4, A and B, green curves). A slight rise in viral loads in samples collected at 3 dpi was detected (at the end of the cohousing), confirming that peptide-treated animals were exposed to SARS-CoV-2. In Fig. 4C, the area under the curve (AUC) shows the notable difference between the mock-treated and the peptide-treated animals. No infectious virus was isolated from lipopeptide-treated ferrets, whereas infectious virus was detected in all mock-treated ferrets (Fig. 4D). Virus isolation data correlated with genome detection (Fig. 4E).

Seroconversion occurred in donor ferrets and six of six mock-treated animals by 21 dpi but occurred in none of the peptide-treated recipient animals, as shown by S- and nucleocapsid (N)specific immunoglobulin G (IgG) enzyme-linked immunosorbent assay (ELISA) and virus neutralization (Fig. 4, F to H). Successful challenge infection confirmed that in-host virus replication had been completely blocked by the [SARS_HRC_-PEG_4_]_2_-chol treatment (Fig. 4I and fig. S8) and that none of the peptide-treated animals were protected, whereas the mock-treated animals (which had seroconverted) were all protected. Collectively, these data show that intranasal prophylactic administration of the [SARS_HRC_-PEG_4_]_2_-chol peptide had protected six of six ferrets from transmission and productive infection.

In light of the persistence of the dimeric lipopeptide in the murine lung (Fig. 2 and fig. S4), we assessed the potential for a single administration of sucrose-formulated lipopeptide in a ferret transmission experiment 2 hours before cohousing to prevent or delay infection. In this experiment, we used a dimeric HPIV-3specific lipopeptide as a mock control (fig. S9). Although sucrose formulation had resulted in promising results in vitro at small scale (Fig. 3B), formulation at larger scale resulted in incomplete dissolution. As a consequence, the sucrose-formulated [SARS_HRC_-PEG_4_]_2_-chol lipopeptide was administered at a substantially lower concentration than in the experiment with the DMSO-formulated lipopeptide (fig. S7, C and D). Nevertheless, the SARS-CoV-2 lipopeptide provided a significant level of protection as compared with the HPIV-3 control group, and four of six SARS-CoV-2 lipopeptide-treated animals were protected against infection. This experiment suggests that single-administration preexposure prophylaxis is promising, although the optimal formulation and dosing regimen are an area of ongoing experimentation.

The intranasal [SARS_HRC_-PEG_4_]_2_-chol peptide presented in this study is a successful prophylaxis that prevents SARS-CoV-2 transmission in a relevant animal model, providing complete protection during a 24-hour period of intense direct contact. Parallel approaches to prevent transmission that target the interaction between S and angiotensin-converting enzyme 2 have shown promise in vitro [e.g., the miniprotein approach (*24*)]. The lipopeptide described here acts on the S2 domain after shedding of S1 (fig. S2 and movie S1) and is complementary to strategies that target the functions of S1 or maintain S in its prefusion conformation, e.g., synthetic nanobodies (*25*). Fusion-inhibitory lipopeptides could be used for pre- and postexposure prophylaxis in combination with these strategies and in conjunction with treatments [e.g., ribonucleoside analogs (*26*)] that reduce replication in a treated infected individual. A combination of drugs that target different aspects of the viral life cycle is likely ideal for this rapidly evolving virus. Of note, the [SARS_HRC_-PEG_4_]_2_-chol lipopeptide is equally active against several emerging SARS-CoV-2 variants, including D614G as well as the recent variants of concerns B.1.1.7 and B.1.351. The [SARS_HRC_-PEG_4_]_2_-chol peptide has a long shelf life, does not require refrigeration, and can easily be administered, making it particularly suited to treating hard-to-reach populations. This is key in the context of COVID-19, which has affected every community, with the burden falling disproportionately on low-income and otherwise marginalized communities. This HRC lipopeptide fusion inhibitor is feasible for advancement to human use and should readily translate into a safe and effective nasal spray or inhalation-administered fusion inhibitor for SARS-CoV-2 prophylaxis, supporting containment of the ongoing COVID-19 pandemic.

## Supplementary Materials

science.sciencemag.org/content/371/6536/1379/suppl/DC1 Materials and Methods Figs. S1 to S10 Table S1 References (*27*,*33*) MDAR Reproducibility Checklist Movie S1

## Appendix

View/request a protocol for this paper from *Bio-protocol*.
